# Early Age Shrinkage and Mechanical Effect of Ultra-High-Performance Concrete Composite Deck: A Case Study with In Situ Test and Numerical Simulation

**DOI:** 10.3390/ma15103628

**Published:** 2022-05-19

**Authors:** Zhiguo Ma, Yue Li, Zhensheng Cao, Shaoqiang Zhang, Shengjun Hou, Jilin Liu, Xin Ruan

**Affiliations:** 1 China Power Construction Group Co., Ltd., Beijing 100037, China; lqmazhg@powerchina.cn (Z.M.); lqcaozs@powerchina.cn (Z.C.); lqzhangsq@powerchina.cn (S.Z.); lqhousj@powerchina.cn (S.H.); 2 Department of Bridge Engineering, Tongji University, Shanghai 200092, China; yueli@tongji.edu.cn (Y.L.); liujilin@tongji.edu.cn (J.L.)

**Keywords:** early age shrinkage, ultrahigh-performance concrete, composite deck, in situ test, numerical simulation

## Abstract

For the Honghe Bridge project located in Yunnan Province, Southwest China, a steel/ultrahigh-performance concrete (UHPC) composite deck is used in the suspension bridge with a 700 m main span, and the steel stud connectors are used in the 50 mm–thick UHPC layer. To investigate the shrinkage behavior of UHPC and the relevant influence, the in situ time-dependent strain is measured continuously, and within the 20-day curing time, the material behavior is summarized based on test results. This paper proposes a prediction model for UHPC shrinkage which is refined from the widely used B3 model for normal concrete material, and the parameter values are modified and optimized by experimental comparison. Combining the numerical model and the finite element analysis model of the composite deck, the detailed mechanical state in structural parts is studied. For the practical construction, the simulation results indicate that the small thickness of UHPC above the stud and weak bond strength can influence the eventual structural performance greatly. In the discussion of stress distribution at different locations of the deck, the potential crack on the edge and the corner of the UHPC–steel interface and the mechanical damage on the stud connector around are also indicated.

## 1. Introduction

For in situ construction, the influence of environmental conditions, such as temperature and relative humidity, is remarkable on structural short-term response and long-term service performance. Focusing on the potential problem related to the featured environment, the engineers and researchers have conducted investigations and proposed solutions to construction specifications. Zhang et al. [1] tested the environment’s influence on cement early age hydration, and the result can guide the control and optimization of construction curing. The structural durability was investigated by Yu et al. [2] in an experimental approach, and the recommendation of decreasing the water-to-binder ratio is proposed to improve the structural life-cycle performance. With improved structural theory and construction techniques, the problems of environmental influence have been treated well with the traditional structural forms and related performance [3,4]. Recently, for the increasing requirement along with the development of quality control and intelligent construction, new materials such as fiber-reinforced-polymer, steel/fiber-reinforced-concrete, and ultrahigh-performance concrete (UHPC) and novel structural forms such as a composite girder and prefabricated column are applied in actual projects [5,6,7,8].

Within the advanced construction materials, the advantages of UHPC are remarkable and, thus, have drawn the attention of researchers and engineers [9]. UHPC has nearly three times the strength of normal concrete and high constructability, and it has been applied in the structural joint, bridge deck, or even the entire pedestrian bridge [10,11,12,13]. However, since they are mixed from different material components, the physicochemical properties of UHPC are quite different from normal construction materials, and the common construction characteristics remain to be investigated, especially the early age shrinkage behavior [14,15,16,17]. Due to the high content of binder and low water-to-cement ratio, the autogenous shrinkage of UHPC is much higher than normal concrete, and with the potential shrinkage crack, the material strength can be reduced over time [18]. Sun et al. [19] focused on the effect of specimen size on the UHPC’s shrinkage behavior. The experimental comparison indicates that the autogenous shrinkage is the predominant mechanism in the total shrinkage of UHPC, and due to the little influence of drying shrinkage, the effect of construction size and environment humidity is not remarkable. The current prediction models for normal concrete are mainly proposed, considering the humidity decreasing and drying shrinkage, which cannot be used in UHPC’s shrinkage directly. To mitigate the effect of autogenous shrinkage, Li et al. [20] and Liu et al. [21] tried adding the MgO, CaO, and other polymer additives as an expansive agent to compensate for the material volume decreasing, but the mechanical strength can be influenced by some agents. Currently, with more and more data on UHPC shrinkage being reported and published, the description and prediction of material long-term shrinkage can be easier, but the continuous investigation of early age behavior is still rare [22,23]. Moreover, different from the shrinkage of plain UHPC or simple structures, the analysis of actual material behavior in complex shapes and boundary conditions can be more difficult, and it is necessary to investigate the detailed mechanical state in engineering applications.

In this paper, the early age shrinkage of steel–UHPC composite deck in the actual structure project, the Honghe Bridge located in the southwest of China, with a 700 m main span, is studied. Firstly, the detailed design of the composite deck is introduced, including material type, stud connector layout, and environmental condition. Secondly, to obtain the actual UHPC shrinkage process, the strain gauge is fixed in situ, and the continuous early age material behavior is measured. Finally, refined from the existing model for normal concrete, the model of UHPC shrinkage is proposed, and the detailed mechanical relationship in the composite deck is analyzed.

## 2. Steel–UHPC Composite Deck in Honghe Bridge Project

**Figure 1:**
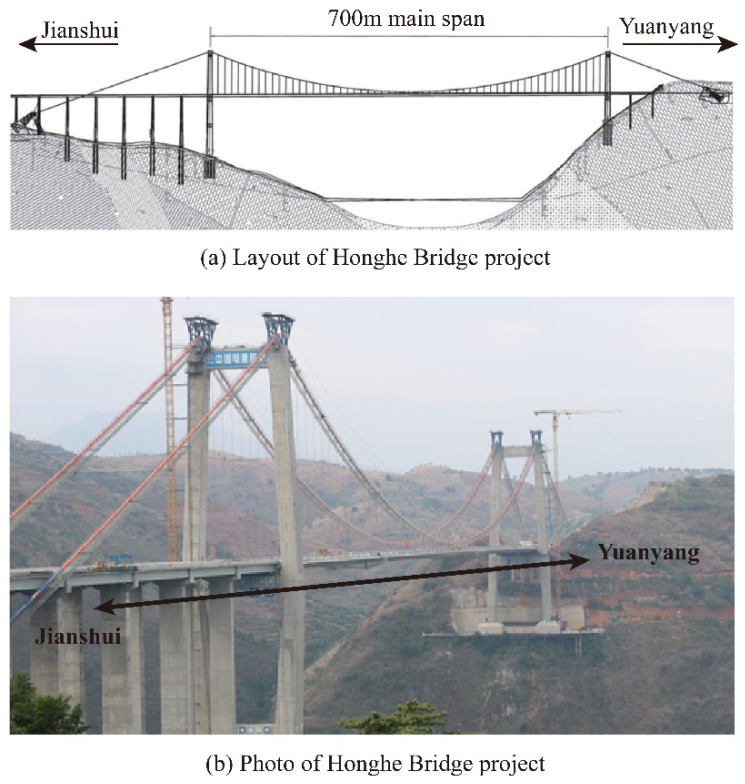
The layout of Honghe Bridge project.

The project in this study is the Honghe Bridge located in Honghe Prefecture, Yunnan Province, Southwest China; it is the critical part of the highway network connecting two counties, Jianshui and Yuanyang. The project is of great significance in regard to boosting the development of Yunnan province and the economic communication with cities around, and the potential in the quick response to natural disasters and the regional poverty reduction is remarkable. The structure is formed in the simply supported suspension bridge with a stiffening steel box girder in a 700 m main span, and the side spans are formed in steel–concrete composite girders and T-girders. The two reinforced-concrete pylons are designed for 181.286 m and 120.486 m. The overall layout of the bridge is shown in Figure 1.

Located in the highland region in Southwest China, the bridge structure is mainly designed by considering the subtropical service environment, but due to the complex terrain, the environments around the structure are quite different vertically. The annual average temperature is 24.4 °C, with the highest temperature at 44.1 °C and the lowest temperature at 3.7 °C, and the annual precipitation is 889.5 mm on average, 1189.1 mm at maximum, and 665.7 mm at minimum. The climate with high temperatures and large precipitation can be of great influence on the bridge construction. The construction of the composite deck of Honghe Bridge was mainly in November and December of 2020. During the curing of the composite deck, the environmental temperature mainly fluctuated from 10 to 25 °C, and the relative humidity was over 50% most of the time. According to the curves, the negative correlation between the environmental temperature and relative humidity is remarkable, especially in the cycles of day and night. At night, the environment temperature drops about 7 °C, and the relative humidity increases to about 85%, whereas the phenomenon is the opposite during the day.

**Figure 2:**
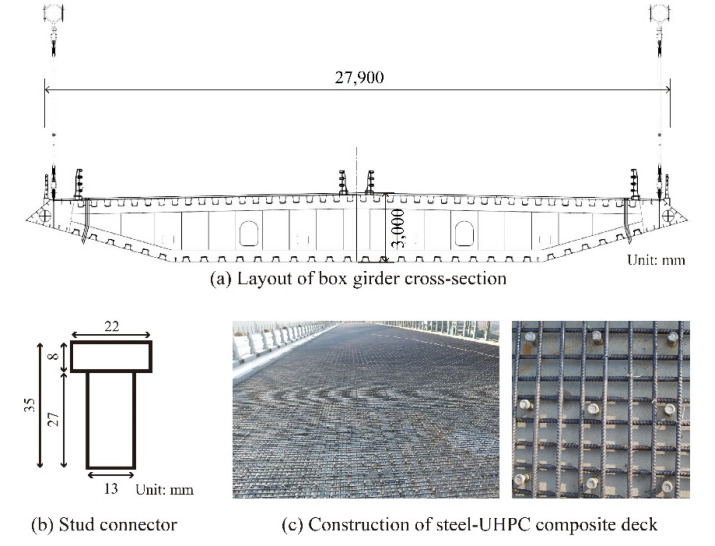
The layout of the girder cross-section, stud connector, and construction of the composite deck.

To bear the vehicle load on the highway and keep the reliable bonding relationship with the steel box girder, the bridge deck is designed in the form of a steel–UHPC composite structure with the stud connector. The type of stud connector that was adopted is 35 mm in height and 13 mm in diameter (shown in Figure 2). The welding layout of the stud connector is set with 150 mm longitudinal and transverse spacing.

**Figure 3:**
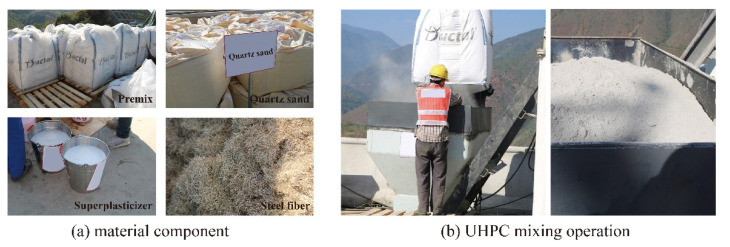
Material component and mixing operation of UHPC.

The Ductal^®^ UHPC product from Holcim Technology, a company belonging to the LafargeHolcim group located in Switzerland, is used in the construction of the composite deck, and the detailed mixing proportion is presented in Table 1. The smooth straight steel fibers are 12.7 mm in length and 0.2 mm in diameter. In the mixing procedure, the premix and quartz sand were mixed for 3 min at first, then the water and the superplasticizer were added and mixed for 5 min, and then the steel fibers were added and mixed for 3 min. The detailed photos and mixing components and construction procedures are presented in Figure 3.

## 3. Experiment and Result

**Figure 4:**
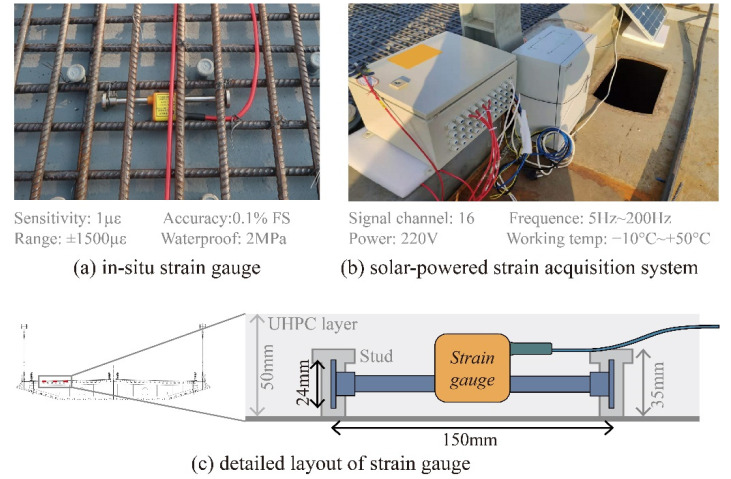
Photo and layout of strain gauge fixed in deck and acquisition system.

To investigate the UHPC shrinkage behavior in the actual composite deck, the strain gauges were fixed between studs before casting. In the in situ experiment, three similar locations in the deck were tested to consider the potential result variation, which can be caused by material heterogeneity or construction operations, and the time-dependent strains were measured every 10 min during the whole casting and hardening process, as shown in Figure 4. To avoid the structural complex effect in the longitudinal direction, such as the construction traffic load or temperature response, the strain gauges were set in the transverse direction. During the curing of UHPC, most shrinkage was completed within the first week, and the material behavior in the first 20 days can reflect the UHPC characteristics to some degree. Based on the measured material behavior, the definition of early age shrinkage in 90 days for normal concrete is changed herein for the rapid shrinkage of UHPC [24]. For the in situ test during the construction of the actual project, the research environment was quite different from the work in the laboratory, so the UHPC shrinkage within 20 days was measured in this study.

**Figure 5:**
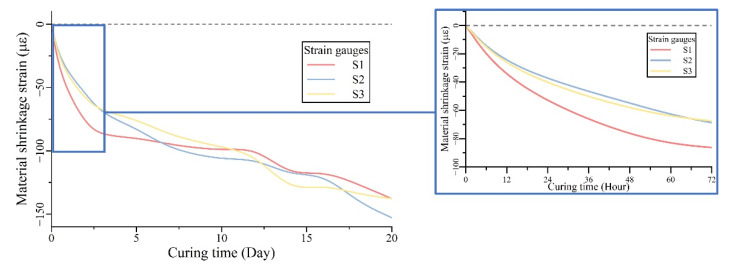
Experimental result of material shrinkage strain in UHPC composite deck.

As the three curves show in Figure 5, the measured strains in three locations have a similar trend and value level. In the first two days after casting, the UHPC material had a rapid shrinkage to around 70 με, and in the following two weeks, the material kept shrinking slowly to about 140 με. The result variation in the shrinkage strains at different locations is relatively small: about 20 με on the second day of curing and eventually reaches about 15 με on the 20th day. When comparing the results of different gauges, the result difference can be caused by material variation, which can be related to the local fiber distribution, orientation, and cementitious matrix hydration. Compared with the measured 140 με shrinkage on the 20th day, the variation in results is still acceptable for the following investigation.

## 4. Numerical Model and Simulation

To investigate the UHPC time-dependent shrinkage behavior and the detailed mechanical response within the composite deck structure, the existing shrinkage and creep prediction B3 or B4 model can be introduced from the research of the concrete material [25,26], which describes the early age shrinkage strain as the time-dependent function influenced by the material properties and the environmental relative humidity. The B4 model is proposed for the study of the short-term and long-term shrinkage of uncracked prestressed concrete girders, as this model contains a series of numerical solutions for detecting creep and shrinkage in uncracked concrete members; however, the B3 model is introduced herein for the simple function form, which can be optimized and promoted conveniently. For UHPC material, the shrinkage mechanism is similar to that of normal concrete, including autogenous shrinkage and drying shrinkage, so the corresponding shrinkage prediction model can also be proposed as presented in Equation (1).

(1) $$\varepsilon_{UHPC}\left(t,t_{0}\right)=-\varepsilon_{UHPC,u}\cdot \varphi_{UHPC}\left(t,t_{0}\right)$$

where $\varepsilon_{UHPC}$ is the shrinkage strain of UHPC material, and $\varepsilon_{UHPC,u}$ is the ultimate shrinkage strain. Due to the little drying shrinkage effect in UHPC, the influence of environmental relative humidity can be ignored. Moreover, $\varphi_{UHPC}\left(t,t_{0}\right)$ is the time-dependent function, which is calculated by the hyperbolic tangent (tanh) function of curing time, t, and initial shrinkage time point, $t_{0}$, and the influence of temperature on curve shape is considered in the environmental coefficient, α, along with the temporal coefficient, $\tau_{UHPC}$. The detailed calculation function is presented in Equation (2).

(2) $$\varphi_{UHPC}\left(t,t_{0}\right)=\tanh\left(\sqrt{\frac{t-t_{0}}{\alpha \cdot \tau_{UHPC}}}\right)$$

where the environmental coefficient, α, is related to the curing temperature, and the temporal coefficient, $\tau_{UHPC}$, is related to the material type.

**Figure 6:**
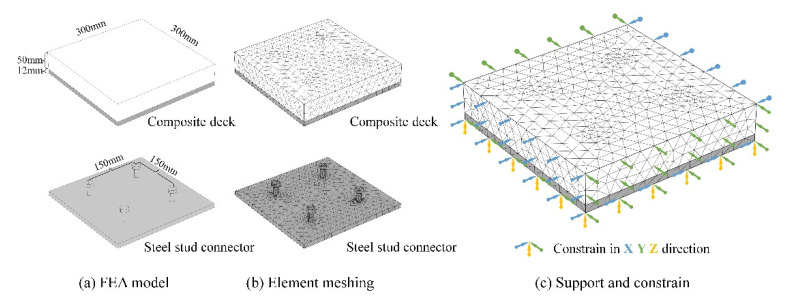
Illustration of FEA model, element meshing, and boundary-condition setting.

According to the actual structure of the steel–UHPC composite deck, the finite element analysis (FEA) model was built to measure 300 mm × 300 mm × 67 mm and followed the same construction details, which include a steel plate that is 12 mm in thickness and a UHPC layer that is 50 mm in thickness. Four stud connectors were built in the model with 150 mm spacing. The UHPC layer, steel plate, and stud connectors were assigned with the corresponding material mechanical properties, respectively. For the shrinkage process of UHPC, the time-dependent strain was forced on the elements within the domain of the UHPC layer, and by the FE solution of mechanical relationship in the composite deck, the actual material behavior and stress distribution could be obtained. Considering that the gravity effect of a composite deck is mainly born by the steel girder structure and is generally not involved in the shrinkage process, the gravity influence was ignored in the simulation reasonably. In the setting of model support and constraint, the rigid constraints were attached on the bottom surface of the steel plate, considering the very little deformation with structural limitation horizontally, and the symmetric boundaries were set on all lateral surfaces. The FEA model and boundary conditions are illustrated in Figure 6, and the boundary conditions in the X, Y, and Z directions are illustrated with arrows in blue, green, and yellow colors, respectively. In the FEA solution, the free tetrahedral element was adopted in model meshing, and for the neighborhood element from different phases, the same nodes were shared on the interface. The maximum meshing size was controlled to be smaller than 10.0 mm, and the FEA model was divided into 154,116 elements.

Focusing on the mechanical relationship between the UHPC layer and the steel structure in the composite deck, the basic material mechanical properties were introduced, and the models were solved in linear analysis with COMSOL software. However, the complex behaviors of UHPC in shrinkage, including nonlinear deformation and local microcrack, are also important and interesting for the following investigations.

**Figure 7:**
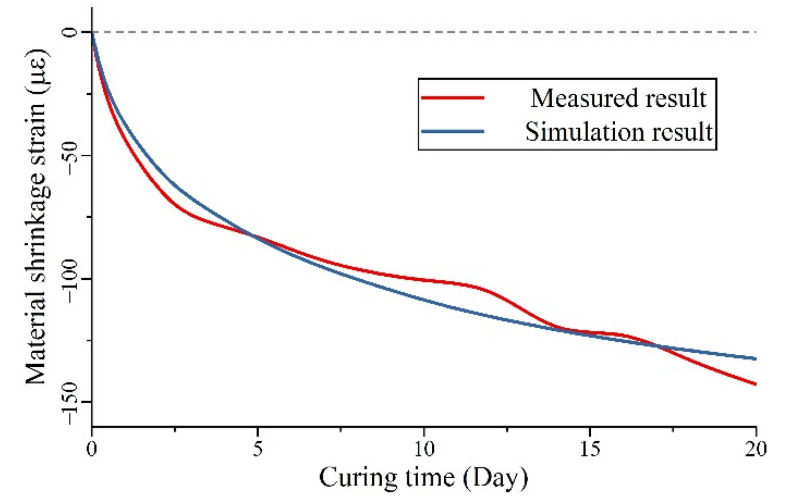
Comparison of measured shrinkage strain and simulation result.

Based on the prediction model and experimental results, the detailed parameter values can be determined by the modification and optimization in the FEA model. With the deformation of two points at the two end locations of strain gauges, the equivalent shrinkage strain in the FEA model can be calculated. With the optimization of model parameters, the eventually regressed parameter values are presented in Table 2, and the comparison of the averaged experimental results and FEA simulation is shown in Figure 7. In the curve comparison, the initialrapid drop in strain after casting and the following trend in measured results are reflected well in the simulated UHPC material. Within the first 10 days, the shrinkage strain difference was controlled under 10 με, and in the following curing days, the difference of shrinkage strain between the experiment and simulation results could also be controlled to around 10 με on the 20th day of curing. Based on the curves shown in Figure 7 and detailed value presented in Table 3, we can see that the actual strain kept slightly decreasing around the 20th day, which can be caused by the different hydration processes of UHPC compared with normal concrete. For the following work, the more advanced numerical model should be introduced specifically for this material. Currently, compared with around 140 με shrinkage in total, the simulation error is smaller than 7.8%, and the accuracy is still acceptable to reflect the mechanical relationship in the composite deck.

**Figure 8:**
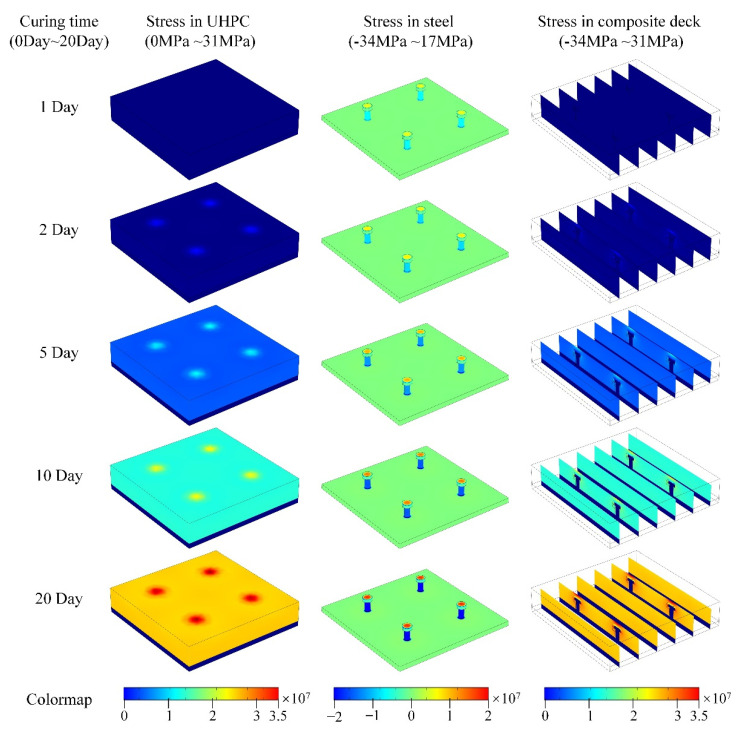
The stress distribution in the UHPC layer, the steel stud connectors, and the model slices in the composite’s deck.

With the FEA model, the time-dependent mechanical response in the composite deck can be predicted conveniently, and the detailed state of UHPC and steel connectors can also be analyzed. As presented in Figure 8, the detailed stress distribution in the UHPC layer and the steel stud connectors after 1-, 2-, 5-, 10-, and 20-day curing were calculated, and the corresponding values are presented in Table 4. In the shrinkage process, the tensile stress in the UHPC layer keeps increasing from 0 MPa to about 20 MPa, due to the limitation of the horizontal direction, which is a uniform distribution. However, the deformation of UHPC around studs is limited, thus leading to the relatively higher tensile stress on the upper surface. The extreme local stress reaches about 31 MPa and raises the risk of local crack greatly. The stress distribution in steel plate is slight tension, which is around 0.04 MPa, but the mechanical state in the studs is relatively complex, and it is explained below. For the mechanical relationship within the whole composite deck, the stress distribution slices of the model are also presented in Figure 8. The decreasing volume in the UHPC causes the vertical deformation in the upper surface, and the studs’ limitation leads to local uplifts and extreme tensile stress.

**Figure 9:**
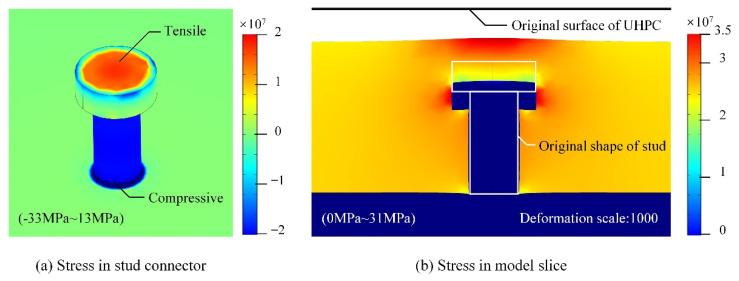
Detailed mechanical relationship with UHPC shrinkage and steel stud connector.

To illustrate the detailed mechanical state in the steel stud connector and the UHPC layer after shrinkage, the detailed stress distributions around a single stud are presented in Figure 9. Due to the UHPC’s uniform tensile stress in the horizontal direction, the head of the stud is also dragged around, and the tensile stress can be found in the center of the stud head. The compression stress in the stud body is mainly caused by the vertical deformation in the UHPC layer, and the local shrinkage of UHPC presses the stud downward on the steel plate, which also is found in the deformation of the steel plate, as the model slice shown in Figure 9b illustrates. The complex stress distribution in the stud is reasonable, as it can point out the potential construction risk that can be caused by UHPC shrinkage. The small thickness of UHPC above the stud can lead to the local crack on the deck’s upper surface, and in the composite deck with a rigid stud or a weak steel–UHPC bond strength, the stud can lift the UHPC layer and influence the structural performance.

## 5. Discussion

**Figure 10:**
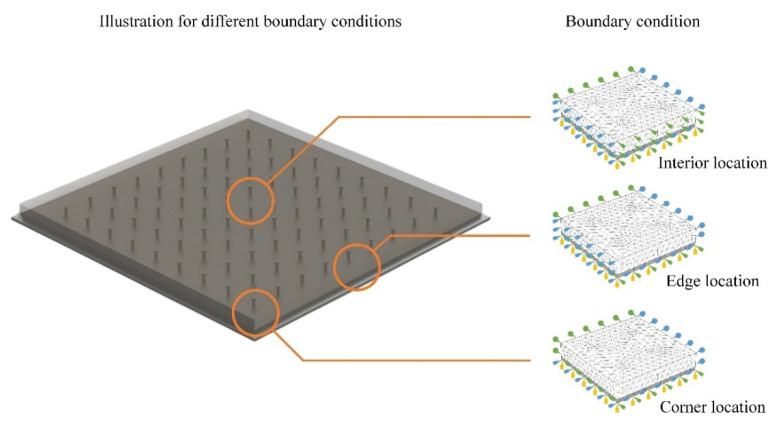
Boundary-condition modification of the FEA model.

In the actual construction of the composite deck, the boundary condition can be various in different locations, and the corresponding mechanical influences can be complex. To analyze the detailed shrinkage behavior in the deck, the constrained settings of the FEA model were modified by considering the interior, edge, and corner location, as shown in Figure 10. The boundary conditions in the X, Y, and Z directions are illustrated with arrows in blue, green, and yellow colors. Compared with the basic model with all lateral surfaces fixed, the model with one or two free surfaces was solved to consider the different shrinkage process in the deck edge and corner, and the other parameters of material properties and shrinkage process were controlled consistently. As seen in the illustration in Figure 10, the model standing for the shrinkage in the internal location are fixed with normal direction constraint in all lateral surface. For the model standing for the edge location, the constraint on one side surface is canceled, and for the model standing for the corner location, the constraint on two neighbor surfaces is canceled.

**Figure 11:**
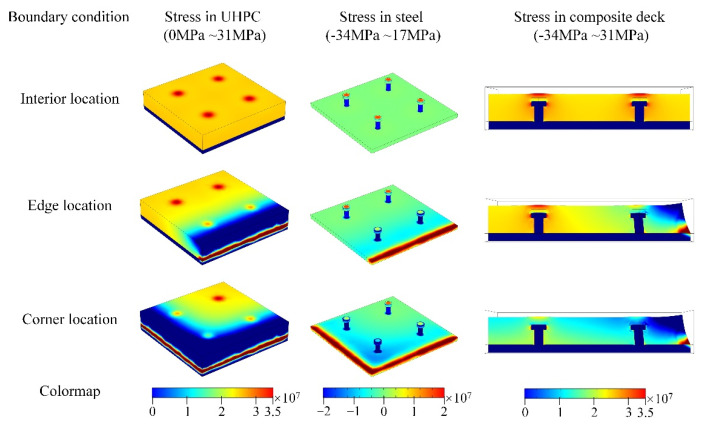
Stress distribution in the edge or corner of UHPC–steel composite deck.

As the simulation results of the three models show in Figure 11, the detailed stress distribution in UHPC and steel studs were calculated, and the influence of different boundary conditions was remarkable. Due to the lack of horizontal constraint, the shrinkage-induced tensile stress was relieved greatly in the edge and corner of UHPC. However, the shearing stress was large around the edge of the UHPC–steel interface, thus indicating the risk of interface crack, and it can be an unexpected transport channel for environmental adverse ions. For the steel plate, the effect of UHPC shearing stress can also be found. For the steel stud, the horizontal shrinkage deformation leads to an inward pushing force on the studs, so the potential mechanical damage on the stud connector should also be concerned in deck construction, especially in the edge or corner. The maximum shearing stresses are relatively high at the edge and corner of the steel–UHPC interface, especially at the edge. Currently, the constitutive model of the material and interface relationship is simple compared with the actual situation, and the stress level can be smaller than simulated, which can be reflected precisely with further model refinement.

## 6. Conclusions

In the Honghe Bridge project, the steel–UHPC composite deck was adopted in the main girder of the suspension bridge structure, and the UHPC’s shrinkage behavior was of great significance for the construction quality control. In the current paper, to investigate the UHPC shrinkage process in this composite deck, the in situ time-dependent strain was measured continuously, and within the 20-day curing time, the material behavior was summarized based on the test result. To study the detailed mechanical state in the UHPC layer and steel members, the prediction model for UHPC shrinkage was proposed and refined on the widely used B3 model, which was modified and optimized by experimental comparison, and the detailed stress distribution in different structural parts was also analyzed. From the tests and numerical simulations, the following conclusion can be drawn.

Measured in the actual steel–UHPC composite deck, the shrinkage process of UHPC consists of two main stages, including the initial rapid shrinkage to around 70 με in the first two days and the slower shrinkage to around 140 με in the following eighteen days. By comparing the results from different gauges, the shrinkage behaviors are similar, which can be used in proposing the prediction model for UHPC shrinkage.

Refined from the existing shrinkage and creep prediction B3 model for concrete material, the model for UHPC shrinkage prediction was proposed herein and considered the influence of the larger ultimate shrinkage and time-dependent process related to the material type. The corresponding parameter values were regressed by the FEA simulation and comparison with the test result, thus making the analysis of the UHPC shrinkage process convenient.

In the discussion of stress distribution in the different locations of the composite deck, the influence of structural boundary conditions is remarkable. The tensile stress is relieved greatly in the edge and corner of the UHPC layer, but the much larger shearing stress can be found on the corresponding UHPC–steel interface. Due to the UHPC horizontal deformation, the pushing forces on the studs around are also indicated in the model comparison. According to the simulation result, the risk of potential interface crack and horizontal mechanical damage on the stud should be concerned in the construction. Based on the current work, the experiment of UHPC shrinkage under different curing environments and the corresponding multiphysics simulation method will also be investigated in the future.

## Figures and Tables

**Table 1:** Mixing proportion of UHPC.

Premix	Quartz Sand	Superplasticizer	Steel Fiber	Water
kg/m^3^	kg/m^3^	kg/m^3^	kg/m^3^	kg/m^3^
1190	1070	38	165	137

**Table 2:** Regressed parameter value after model modification.

**Model Parameter**	$\varepsilon_{UHPC,u}$	$\alpha \cdot \tau_{UHPC}$
**Unit**	με	Day
**Value**	161	14.738

**Table 3:** Prediction error of numerical model compared with measured results.

Curing Time	Measured Result	Numerical Model	Absolute Error	Relative Error
Day	με	με	με	%
1	−40.1	−41.0	0.9	2.2
2	−64.7	−56.8	8.0	12.4
5	−82.1	−84.4	2.3	2.8
10	−101.4	−109.0	7.6	7.5
15	−125.7	−123.2	2.5	2.0
20	−142.8	−132.4	10.3	7.2

**Table 4:** Stress value in UHPC and steel during shrinkage process.

Curing Time	Stress in the UHPC Layer		Stress in Steel Plate and Studs	
	Min Value	Max Value	Min Value	Max Value
Day	MPa	MPa	MPa	MPa
1	6.8	9.9	−11.1	4.5
2	8.7	12.8	−14.3	5.7
5	12.0	17.8	−20.2	8.1
10	15.5	22.9	−26.1	10.5
20	19.9	29.4	−33.7	13.5
